# Simultaneous biportal endoscopic management of pineal region tumors in patients with obstructive hydrocephalus: technical notes

**DOI:** 10.1186/s41016-022-00313-0

**Published:** 2023-01-10

**Authors:** Sultan Al-Saiari, Khalid Al Orabi, Mohammad Ghazi Abdoh, Abdulaziz A. Basurrah, Sultan Faez Albalawi, Ahmed A. Farag

**Affiliations:** Neurosurgery Department, King Abdullah Medical City, P.O 24246, Makkah, Saudi Arabia

**Keywords:** Pineal tumors, Germinoma, Hydrocephalus, Endoscopic third ventriculostomy, Endoscopic biopsy

## Abstract

**Background:**

The goal of this study is to show the feasibility and benefits of using the simultaneous biportal endoscopic procedure to treat pineal tumors in patients with obstructive hydrocephalus.

**Methods:**

We retrospectively reviewed three patients with pineal tumors and acute obstructive hydrocephalus who were treated in one session with a frameless stereotactic guided simultaneous biportal endoscopic third ventriculostomy and endoscopic tumor biopsy performed through two separate ports using one rigid ventriculoscope.

**Results:**

In the three patients, ventriculostomy and endoscopic biopsies were conducted. There was no death or morbidity throughout the 45-min procedure. All of the patients’ histological findings were confirmed. Germinoma was diagnosed in two patients who recieved postoperative radiotherapy, and the third patient diagnosed with a pineocytoma. Magnetic resonance imaging with flow-sensitive sequences was used to confirm ventriculostomy patency in all patients 6 months after the surgery.

**Conclusion:**

Biportal endoscopic approach enables better visual control of both procedures. Furthermore, it allows the surgeon to safely pass the ventriculoscope via the foramen of monro, even if it is narrow. Moreover, during endoscopic tumor biopsy and third ventriculostomy, the intracranial pressure can be smoothly managed using the outlet tubes accessible. This treatment may be an alternative to traditional uniportal endoscopic operations in certain patients.

## Background

Pineal tumors account for between 0.6 and 0.9% of all brain tumors [1, 2]. The differential diagnosis for a tumor in this area is quite variable [3]. Germ cell tumors account for 20 to 37% of cases, with intrinsic pineal tumors accounting for 22 to 27%, gliomas accounting for 24 to 28%, and the other lesions accounting for 12 to 32% [4, 5].

Due to the obvious histologic variability, the therapy and prognosis differ, and as a result, the pathological diagnosis is critical [6]. The major goal of primary care is to lower intracranial pressure and determine the tumor’s histological type [7].

Direct removal of such tumors and the trial of restoring the normal CSF channel may expose the patient to a very high risk of complications, especially if the foramina of Monro are not expanded [8]. Furthermore, a high percentage of tumors in this site are radiosensitive, obviating the need for craniotomy [9].

Histopathological data can then be obtained with a less invasive stereotactic biopsy [10], although this will not resolve the hydrocephalus on its own. CSF shunting had the highest rate of problems in these instances, with shunt obstruction being the most common [11, 12]. This is likely due to the high protein concentration of CSF.

As a result, the introduction of neuroendoscopy has provided an efficient means of fulfilling both objectives. In the treatment of obstructive hydrocephalus in those individuals, third ventriculostomy has become a standard procedure and a viable alternative to ventriculo-peritoneal shunt [13]. More advanced approaches have recently been recommended to combine ETV and endoscopic biopsy with either one or two trajectories, utilizing either a rigid or flexible endoscope or both [14–17].

The operating details and benefits of using two trajectories at the same time with two independent entry points for the management of lesions in this area are presented in this study.

## Methods

### Case illustrations

**Fig. 1:**
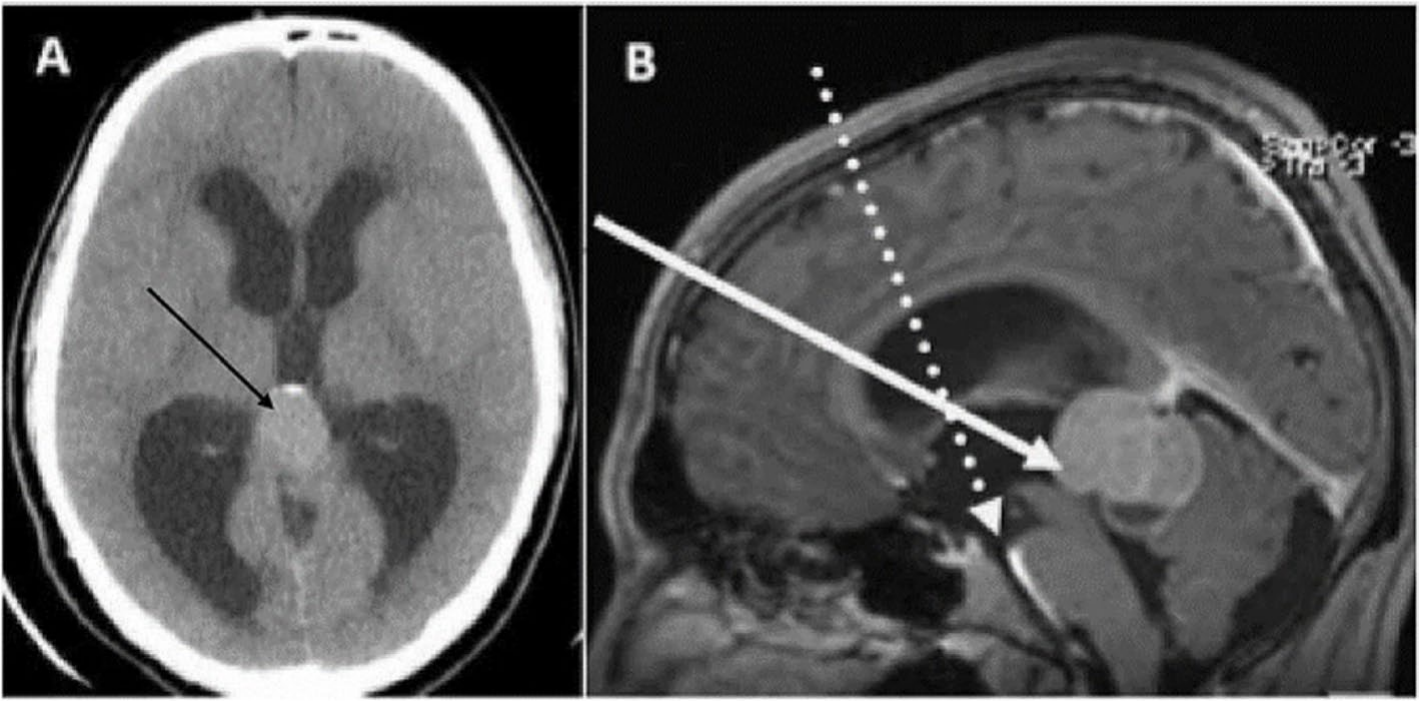
**A** Preoperative plain CT brain showing the lesion (black arrow). **B** Preoperative sagittal T1 MRI with contrast showing the planned endoscopic trajectories. The first trajectory was for third ventriculostomy (dashed arrow) (via the right lateral ventricle and the right foramen of Monro towards the target point in the midline of the floor of the third ventricle, 3 mm in front of the mamillary bodies), and the second trajectory (solid arrow) was for tumor biopsy (via the right frontal horn and the right foramen of Monro towards the midpoint of the tumor)

An 18-year-old man was presented to emergency department with a complaint of an acute headache of a 3-week duration which was worsen over the last 3 days. There was no history of seizures, weakness, or sensory disorders. On examination, he was neurologically intact, apart from grade II papilleodema on ophthalmoscopy. His CT revealed supratentorial obstructive hydrocephalus caused by a solid mass with focal peripheral calcification, in the pineal region (Fig. 1A). Consequently, MRI with contrast was done (Fig. 1B) and revealed lobulated mass identified at the pineal region, overall measures approximately 4 × 3.6 × 2.6 cm in maximum AP, CC, and T dimensions, respectively. It demonstrated elements of mild diffusion restriction and predominant heterogeneously enhancing solid low T2/T1 signal intensity with associated small cystic/necrotic component inferiorly that measures about 17 × 15 mm peripherally located punctate magnetic susceptibility artifacts related to calcifications were reported. The lesion was closely related to the deep cerebral veins with no definite thrombosis or occlusion. It was causing effacement of the quadrigeminal cistern, supracerebellar cistern, and the aqueduct of Sylvius and anteriorly extending to the posterior third ventricle. There was mild compressive mass effect on the brain stem and the cerebellum. Subsequently, it was contributing to moderate severe obstructive hydrocephalous with associated subependymal permeation. No midline shift or herniation were reported. An MRI-navigation-guided endoscopic operation was indicated.

### Operative techniques

**Fig. 2:**
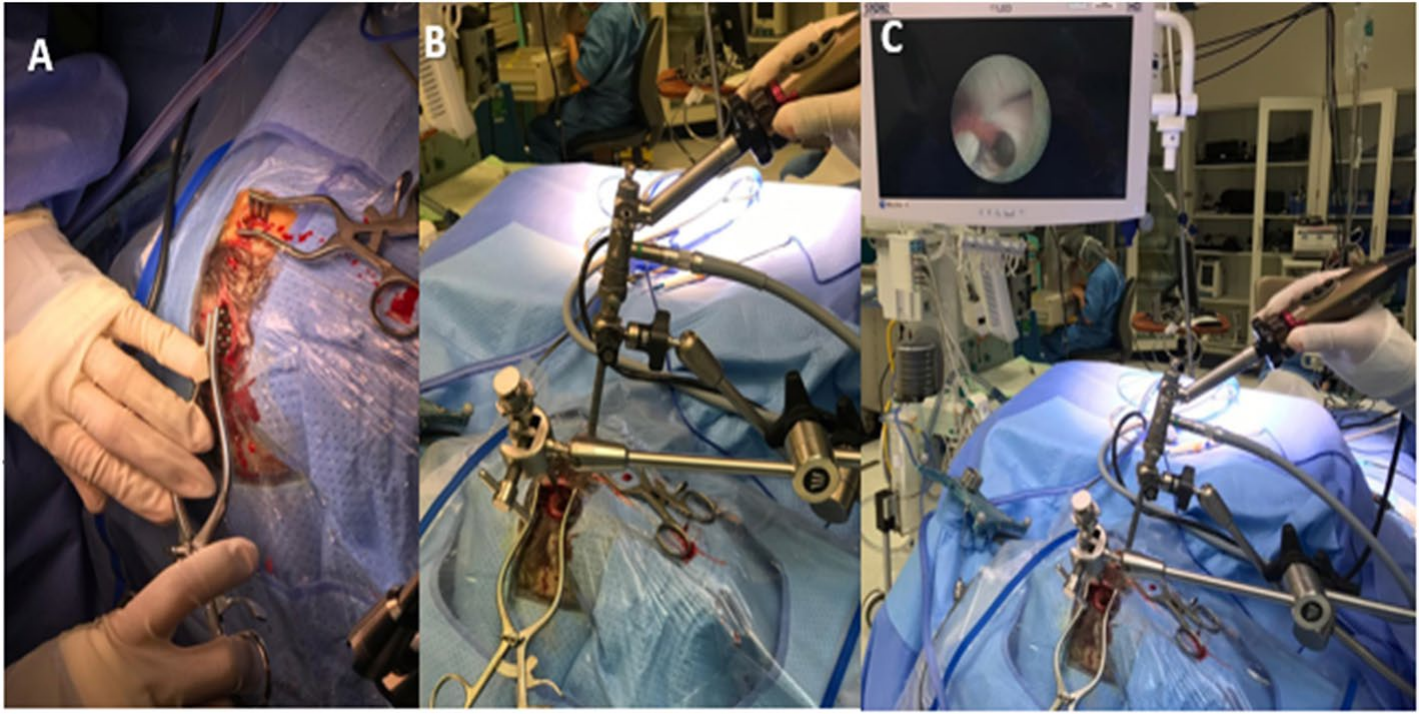
**A** two burr hole. **B**, **C** Two endoscopes fixed in mechanical holder

**Fig. 3:**
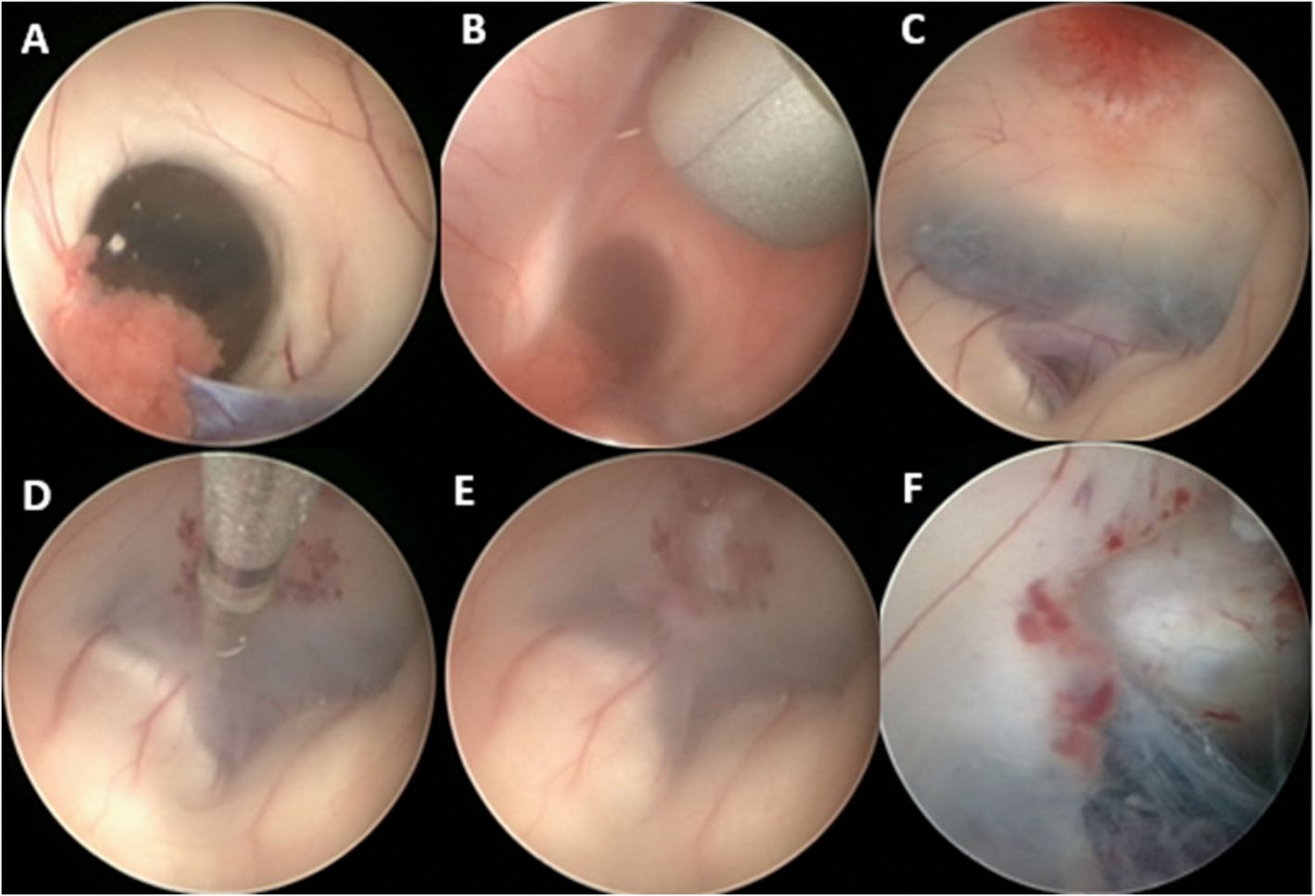
**A**, **B** Operative view through the second endoscope while advancing the first one into foramen of monro. **C**–**F** Operative view through the first ventriculoscope. **C** Floor of 3rd ventricle. **D** Endoscopic forceps performing the ostium. **E** The ventriculostomy opening. **F** Liliequist membrane after opening

**Fig. 4:**
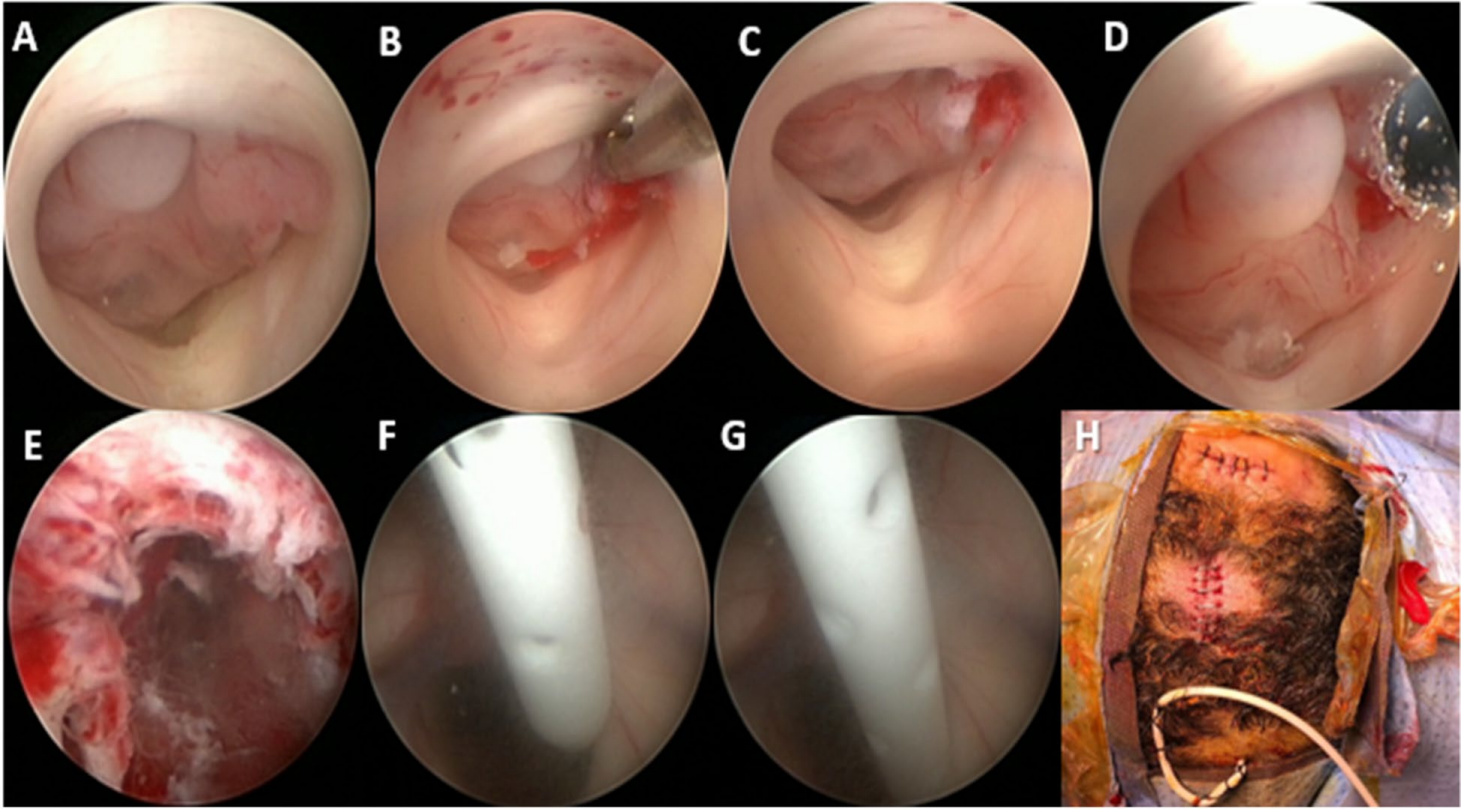
Operative view through the second endoscope, the tip of which is positioned in the posterior part of third ventricle. **A** A large reddish tumor is visualized. **B** Biopsy is taken by endoscopic forceps. **C** Bleeding from biopsy site. **D** Hemostasis by monopolar. **E** Biopsy site after hemostasis. **F**–**G** An EVD is inserted under guidance of the second ventriculoscope. **H** The site of the 2 burr holes

**Fig. 5:**
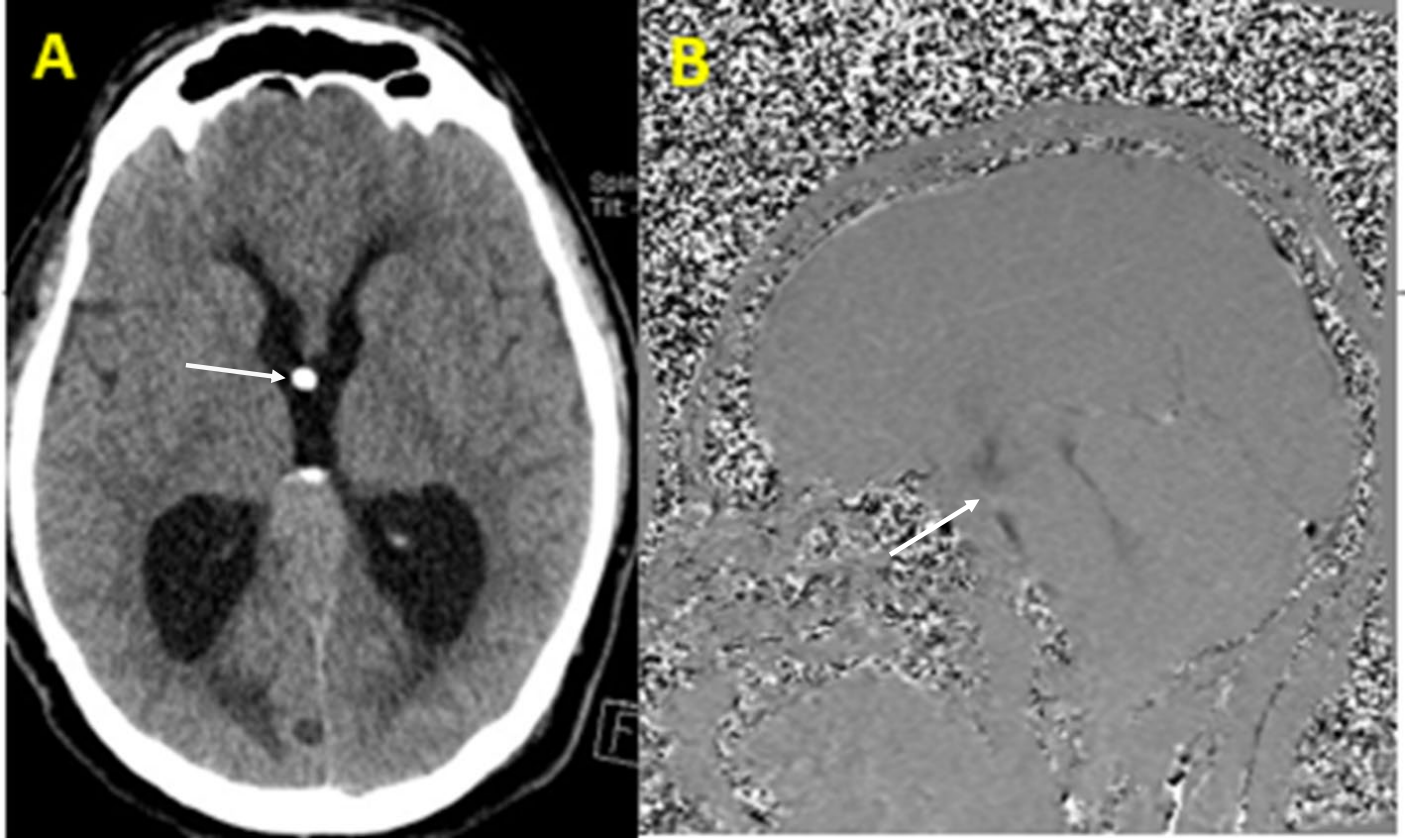
**A** Postoperative CT brain showing the EVD tip (white arrow) passing through foramen of monro. **B** A flow-sensitive MRI showing patent ventriculostomy ostium (white arrow) black signal in MRI with CSF flowmetry due to turbulance of CSF across the ventriculostomy site

Under general anesthesia, the patient was positioned supine on horse shoe which should be as near as possible to operating table in order to make the endoscopic sheaths, attached to the holding arms, reach our targets easily without any minimal tension, the head elevated at 30° and slightly flexed. The proximal holding arm is used for the first trajectory and the distal one for the second trajectory. After registration of the patient, two trajectories were marked by the mean of navigation. The first trajectory was for third ventriculostomy (Fig. 1B dashed arrow) (pre-coronal burr hole and 2.5 cm lateral to MEDLINE, via the right lateral ventricle and the right foramen of Monro towards the target point in the midline of the floor of the third ventricle, 3 mm in front of the mamillary bodies), and the second trajectory (Fig. 1B solid arrow) was for tumor biopsy (posterior to hair line if it is possible and 2.5–3 cm lateral to MEDLINE, via the right frontal horn and the right foramen of Monro towards the midpoint of the tumor (Figs. 2B, C and 3H). After routine preparation of the surgical site, two endoscopic mechanical holders were applied on the operative table (ipsilateral to surgical side, in our cases on the right side of the table). Two burr holes were made over the planned trajectories. The right frontal horn was tapped, using the operating sheath with obturator, stopped just when it passed the ependymal wall of lateral ventricle, then the obturator was changed for a video camera-equipped endoscope (outer diameter, 2.3 mm). CSF was taken for cytological and biochemical analyses. The same steps were applied on the other trajectory with new sheath and obturator and the same rigid endoscope. After confirmation that we are in lateral ventricle, we reput the obturator again and we come back to the first trajectory to realize the ETV and we always start with ETV first in order to avoid any bleeding could happen from the tumor. The ventriculoscope was advanced through opening of the foramen of Monro (Fig. 3B). Opening of the floor of the third ventricle in the midline just in front of the mamillary bodies was performed with endoscopic forceps through the working channel under pressure-controlled constant irrigation via the irrigation channels (Fig. 3C–F). The opening of the ventricle floor was enlarged by inflation of a 3-French Fogarty catheter. Further advancement of the endoscope was performed to open The Liliequist membrane (Fig. 3F). CSF flow towards the basal cisterns and pulsation of the floor of the third ventricle were immediately observed. The ventriculoscope was then withdrawn to the lateral ventricle, the obturator was introduced again into the sheath, and the endoscope was moved to the 2nd trajectory in order to take a biopsy. The damage to the structures of the foramen could be avoided by the straight trajectory when the second burr hole was put on or just posterior to hairline. In the posterior part of third ventricle, a large reddish tumor was visualized (Fig. 4A). Multiple endoscopic biopsies were taken by tumor forceps and hemostais was achieved by monopolar and controlled irrigations with ringer’s lactate solution (Fig. 4B–E). As the possibility of tumors with mixed histology existed, attempts were made to obtain a sufficient quantity of tissue for pathological investigation. It is worth mentioning that there were no recorded changes in heart rate or blood pressure throughout the procedure, which may be due to the multiple outlet channels provided by both endoscopic sheaths, which allow drainage of the irrigating fluid without affecting the physiological intracranial pressure. Once we get a confirmation of the frozen section histopathology and we reach good hemostasis and clear CSF, the obturator removed from first trajectory endoscopic sheath and the EVD was introduced under direct endoscopic vision of the second trajectory, the sheath then was removed. Finally, we remove the whole endoscope from the second trajectory and we cover the burr holes with small pieces of gelfoam and skin was closed. The EVD was kept closed. No further measures were taken. The patient was mobilized on the next day and postoperative CT was done (Fig. 5A) and showed improvement in ventricular size; as a result, the EVD was removed and patient discharged from the hospital on the 3rd day. He made an uneventful recovery, and a flow-sensitive MRI 1 week later confirmed ventriculostomy patency (Fig. 5B).

## Discussion

The goal of treatment for pineal tumors is to remove the tumor while restoring the CSF routes. Before defining a therapy protocol, several significant factors should be examined [7]. Because a large percentage of the tumors are radiosensitive, surgical excision is not required. Infiltrative tumors can be surgically removed, but only in part. Increased intracranial pressure caused by obstructive hydrocephalus can sometimes appear before the symptoms and signs of localized tissue compression. If the intracranial pressure is regulated by internal CSF drainage without any additional steps to lower the tumor size, individuals with benign slow-growing infiltrating neoplasms may endure a long and largely symptom-free time [15]. Direct excision of tumors (transcallosal transventricular transchoroidal, supracerebellar-infratentorial, or transcomissural technique) and restoration of the CSF pathway can result in a high risk of morbidity, particularly if the foramina of Monro are narrowed [17]. Alternative treatments include CSF shunting, performance imaging-guided biopsies, and radiation. However, such instances may show the highest frequency of CSF shunting problems, namely shunt obstruction, which is likely owing to the high CSF protein level. Image-guided biopsy is a separate operation that carries additional hazards, especially in vascularized tumors in such location of crowded area with vascular and brain stem structures [11, 12]. Endoscopic ventriculostomy has risks that are not comparable to shunting operations [15–18].

According to a literature study, the long-term success rate of endoscopic ventriculostomies in cases with secondary aqueduct stenosis is greater than 80%. However, because of the possibility of CSF malabsorption via arachnoid villi, this approach has been shown to be ineffective in non-tumoral aqueductal stenosis, particularly in young infants. As a result, individuals with tumoral aqueductal stenosis are treated as a distinct entity, with endoscopic ventriculostomy being the treatment of choice [18].

Endoscopy not only solves the CSF route obstruction, but it also enables for the determination of the lesions’ histopathological type in a single session [3].

The biportal endoscopic approach allows for a tumor biopsy and quick restoration of physiological CSF routes. Under direct vision, a tumor biopsy can be conducted. as a result, it aids in the prevention of vascular tumor damage. Bimanual-like manipulations are possible with the biportal technique. Furthermore, it provides for the reasonably precise assessment of anatomic structure sizes and distances [17].

During ventriculoscopic procedures, anesthesiologists have recorded intraoperative hemodynamic changes. Hemodynamic shifts are common and usually transient. However, if tachycardia, bradycardia, hypertension, hypotension, or arrhythmia is detected, surgeons should cease fluid irrigation, ballooning, and endoscopic advancement. Intraoperative cardiac arrest has been recorded on a few occasions [19]. The biportal technique permits appropriate drainage of the irrigating fluid and easy management of the pressure in the ventricles via the two irrigation channels of the endoscope through one sheath and another separate sheath of the other trajectory; thus, even if one or more outflow channels are obstructed, all of these concerns are theoretically reduced.

Several publications have recommended drilling one “compromised” burr-hole in the middle of the two optimum entry sites [15]. However, there are some drawbacks, such as increased fornix pressure during ETV and the posterior border of the foramen of Monro during endoscopic biopsy. Furthermore, this approach is only appropriate for big tumors that are not too far posterior [11].

Endoscopic biopsy and third ventriculostomy can be performed with a rigid and flexible endoscope in a single session monoportal method [11]. However, the flexible endoscopic system’s lower optical quality has become a significant restriction, potentially affecting the ability to detect tumor spread [19]. Furthermore, the flexible forceps’ reduced size compared to rigid endoscope forceps may impact the size of the biopsy sample, resulting in inconsistent histology results [20]. Furthermore, maneuvering the flexible ventriculoscope via the foramen of Monro and directing it toward the third ventricle’s massa intermedia can be difficult. Moreover, the tumor should be biopsyed and coagulated without irrigation. More significantly, any bleeding from the biopsy site could result in total vision loss [11].

Only three cases with pineal region lesions, operated through simultaneous biportal techniques, have been reported in the literature up to our knowledge; however, the surgeon performed a CT-guided endoscopic procedure using an imaging-compatible Cosman-Roberts-Wells stereotactic coordinate frame (Radionics), which may be time consuming and technically demanding in comparison to frameless navigation. In addition, he used two endoscopes at the same time through the two trajectories [17].

In all our three patients, there was no surgery-related mortality or morbidity. Flow-sensitive MRI revealed patent ventriculostomies in all of the patients. In two patients with germinoma, postoperative irradiation was performed, and in the third patient with pinocytoma, he refused additional surgical procedures and radiotherapy.

## Conclusions

Simultaneous biportal endoscopic technique allows the surgeon to securely slip the ventriculoscope via the foramen of monro, even if it is narrow. Furthermore, during endoscopic tumor biopsy and third ventriculostomy, the intracranial pressure can be smoothly managed using the multiple outlets accessible. This treatment may be an alternative to traditional uniportal or non-simultaneous biportal operations in certain individuals and well-equipped locations.

Our current study’s main limitation is the small number of patients. More cases are needed to assess the technique’s safety and efficacy in comparison to the traditional techniques.

## Data Availability

Data sharing not applicable to this article as no datasets were generated or analyzed during the current study.

## References

[1] Apuzzo ML, Chandrasoma PT, Cohen D, Zee CS, Zelman V (1987). Computed imaging stereotaxy: experience and perspective related to 500 procedures applied to brain masses. Neurosurgery..

[2] The Committee of Brain Tumor Registry of Japan. Report of brain tumor statistics in Japan. Neurol Med Chir. 2003:1–111 https://pubmed.ncbi.nlm.nih.gov/14705327/. 14705327

[3] Morgenstern PF, Souweidane MM (2013). Pineal region tumors: simultaneous endoscopic third ventriculostomy and tumor biopsy. World Neurosurg..

[4] Konovalov AN, Pitskhelauri DI (2003). Principles of treatment of the pineal region tumors. Surg Neurol..

[5] Regis J, Bouillot P, Rouby-Volot F, Figurearella-Branger D, Dufour H, Peragut JC (1996). Pineal region tumors and the role of stereotactic biopsy: review of the mortality, morbidity, and diagnostic rates in 370 cases. Neurosurgery..

[6] Gangemi M, Maiuri F, Colella G, Buonamassa S (2001). Endoscopic surgery for pineal region tumors. Minim Invasive Neurosurg..

[7] Tamaki N, Yin D (2000). Therapeutic strategies and surgical results for pineal region tumours. J Clin Neurosci..

[8] Rhoton AL, Yamamoto J (1981). Peace AD microsurgery of the third ventricle: part II-operative approaches. Neurosurgery..

[9] Sawaya R, Hawley DK, Tobler WD, Tew JM (1990). Chambers AA: Pineal and third ventricle tumors and in Youmans JrNeurological Surgery.

[10] Roth J, Constantini S (2015). Combined rigid and flexible endoscopy for tumors in the posterior third ventricle. J Neurosurg..

[11] Basauri L, Zuleta A (1982). Shunts and shunt problems. Monogr Neural Sci..

[12] Cedzich C, Kaden B, Schramm J (1992). Pre-and post-operative hydrocephalus in supratentorial interventricular tumours. Acta Neurochir (Wien)..

[13] Schroeder HW (2013). Intraventricular tumors. World Neurosurg..

[14] Oppido PA, Fiorindi A, Benvenuti L, Cattani F (2011). Neuroendoscopic biopsy of ventricular tumors: a multicentric experience. Neurosurg Focus.

[15] Shono T, Natori Y, Morioka T, Torisu R (2007). Results of a long-term follow-up after neuroendoscopic biopsy procedure and third ventriculostomy in patients with intracranial germinomas. J Neurosurg..

[16] Morgenstern PF, Souweidane MM (2013). Pineal region tumors: simultaneous endoscopic third ventriculostomy and tumor biopsy. World Neurosurg..

[17] Veto F, Horváth Z, Dóczi T (1997). Biportal endoscopic management of third ventricle tumors in patients with occlusive hydrocephalus: technical note. Neurosurgery..

[18] Kulkarni AV, Drake JM, Kestle JR, Mallucci CL, Sgouros S, Constantini S (2010). Predicting who will benefit from endoscopic third ventriculostomy compared with shunt insertion in childhood hydrocephalus using the ETV Success Score. Clinical article. J Neurosurg Pediatr.

[19] Chibbaro S, Di Rocco F, Makiese O, Reiss A (2012). Neuroendoscopic management of posterior third ventricle and pineal region tumors: technique, limitation, and possible complication avoidance. Neurosurg Rev..

[20] Kawsar KA, Haque MR, Chowdhury FH (2015). Avoidance and management of perioperative complications of endoscopic third ventriculostomy: the Dhaka experience. J Neurosurg..

